# French recommendations for the management of Takayasu’s arteritis

**DOI:** 10.1186/s13023-021-01922-1

**Published:** 2021-07-21

**Authors:** David Saadoun, Alessandra Bura-Riviere, Chloé Comarmond, Marc Lambert, Alban Redheuil, Tristan Mirault, Collaborators, Paul Achouh, Florence Aeschlimann, Jean-Marc Alsac, Laurent Arnaud, Ygal Benhamou, Boris Bienvenu, Eric Bodiguel, Olivia Boyer, Laurent Chiche, Joel Constans, Raphael Darbon, Sandrine Guibaudet, Philippe Lamarche, Séverin Ferrand, Olivier Espitia, Stephanie Franchi, Julien Gaudric, Pascal Giordana, Eric Hachulla, Pierre-Yves Hatron, Arnaud Hot, Isabelle Kone-Paut, Hélène Maillard, Arsène Mekinian, Pierre Quartier, Thomas Quemeneur, Mickael Soussan, Marc Sapoval, Jean Schmidt, Gilles Soulat

**Affiliations:** 1 Internal medicine, Pitié Salpêtrière Hospital, Paris, France; 2 Vascular medicine, CHU Toulouse, Toulouse, France; 3 Internal medicine, CHU Lille, Lille, France; 4 Vascular radiology, Pitié Salpêtrière Hospital, Paris, France; 5 Vascular medicine, Hopital European Georges Pompidou, Paris, France; 6 Cardiac Surgery, Paris, France; 7 Pediatrician, Paris, France; 8 Vascular Surgery, Paris, France; 9 Rheumatology, Strasbourg, France; 10 Internal Medicine, Rouen, France; 11 Internal Medicine, Marseille, France; 12 Neurology, Paris, France; 13 Pediatric Emergency Medicine, Paris, France; 14 Vascular Medicine, Bordeaux, France; 15 France Vasculitis Association, Paris, France; 16 Internal Medicine, Nantes, France; 17 Pediatric Radiology, Paris, France; 18 Vascular Medicine, Nice, France; 19 Internal Medicine, Lille, France; 20 Internal Medicine, Lyon, France; 21 Pediatrics, Paris, France; 22 Internal Medicine, Paris, France; 23 Pediatrics, Paris, France; 24 Internal Medicine, Valenciennes, France; 25 Nuclear Medicine, Paris, France; 26 Interventional Radiology, Paris, France; 27 Internal Medicine, Amiens, France; 28 Vascular Radiology, Paris, France

## Abstract

The aim of this National Diagnostic and Care Protocol (PNDS) is to explain to the professionals involved the current optimal diagnosis and therapeutic management and care approach for a patient with Takayasu’s arteritis. Its purpose is to optimize and harmonize the management and follow-up of this rare disease throughout the country. It also identifies pharmaceutical specialties used in an indication not provided for in the Marketing Authorization, as well as the specialties, products or services necessary for the care of patients but not usually paid for or reimbursed.

## Summary for the attending physician

This summary was developed from the National Diagnostic and Care Protocol (PNDS) - Takayasu’s Arteritis, available on the website of the French National Health Authority https://www.has-sante.fr/.

Takayasu’s arteritis is vasculitis of the large arterial trunks. This disease occurs before the age of 50 and mainly affects women from the Mediterranean area, Southeast Asia and the Middle East, and it can in rare cases begin at the pediatric age. This inflammatory vascular disease is most often located in the aorta and its cervical, visceral and lower limb branches. It is complicated by aneurysms or arterial stenosis.

Diagnosis of Takayasu’s arteritis should be clinically suspected in the presence of neck pain along the path of the carotid arteries, lack of pulses in the upper limbs, vascular murmurs, claudication of the upper or lower limbs in a young subject. Indirectly, a diagnosis of Takayasu’s arteritis can be considered in view of unusual manifestations, such as high blood pressure in a young subject, a change in the overall condition, episcleritis, erythema nodosum. The inflammatory syndrome is common in the early onset of the disease and should be investigated by the C-reactive protein (CRP) assay and the fibrinogen level or sedimentation rate, which will later be used to monitor the activity of the disease being treated.

The diagnosis of Takayasu’s arteritis is based on a series of data including age, gender, clinical history, and imaging that evidences damage to the aorta and/or its branches with circumferential thickening of the arterial wall, stenosis, and sometimes aneurysmal dilation, often multifocal, and the opinion of a doctor who is an expert on Takayasu’s arteritis.

Oral corticosteroid therapy is the standard treatment for Takayasu’s arteritis. It is introduced at the initial dose of 0.5 to 1 mg/kg/day of oral prednisone in an adult. Subsequently, the corticosteroid therapy is gradually reduced under clinical, biological, and imaging supervision, at best by alternating consultations with the general practitioner and the vascular doctor, internist or rheumatologist with expertise in this disease. The goals for reducing prednisone are to reach a dose of less than 20 mg/day (0.5 mg/kg/day in children) at the end of the third month, and less than 0.1 mg/kg/day (0.25 mg/kg/day in children) at the end of the sixth month. Discontinuing the general corticosteroid therapy should be considered after 24 months of treatment, making sure that there is no secondary adrenal insufficiency. Comorbidities, especially those that would suggest poor tolerance of the corticosteroid therapy, should be investigated. Measures related to the corticosteroid therapy, especially preventing osteoporosis, should also be implemented, at a minimum through vitamin D and calcium supplements. Seasonal pneumococcal and flu vaccinations are recommended. Metabolic tolerance of the treatment, particularly in terms of weight and glycemia, must be evaluated. Prescribing antiaggregant doses of aspirin (75–300 mg/day, 3–5 mg/kg/day in children, not to exceed 75 mg/day) should be considered for patients, if there is no contraindication. High blood pressure should be checked and an antihypertensive treatment should be initiated as soon as necessary (blood pressure should be monitored in the limb where it is highest to avoid adapting the treatment to underestimated pressure values downstream of arterial stenosis).

Regular clinical, biological and imaging monitoring is necessary for patient follow-up, especially to assess the response to the treatment, diagnose relapses and recurrences, manage adverse effects of the corticosteroid therapy and detect delayed vascular complications. Experts suggest putting immunosuppressants (i.e.methotrexate) systematically on as the first line for the purpose of reducing the use of steroids. The immunosuppressants for which there is the most data are methotrexate. Biotherapies have also been shown to be effective in these situations, including anti-TNF-alpha (infliximab, adalimumab, etanercept) and anti-IL6 (tocilizumab). In cases of absolute intolerance to the corticosteroid therapy or difficulties withdrawing from the corticosteroid therapy, taking immunosuppressants, including methotrexate, should be discussed with a doctor who is an expert in Takayasu’s arteritis.

All health professionals and patients should be informed about the existence of patient associations.

## 1 Introduction

### 1.1 Terminology and classification

Takayasu’s arteritis is an anatomo-clinical condition that is part of the vasculitis group. Other terminology used in the past is pulseless disease or aortic arch syndrome. Takayasu’s arteritis is a type of vasculitis affecting the large-caliber vessels, including the aorta and its main branches (subclavian, carotid, vertebral, renal, digestive and iliac arteries), but also the coronary and pulmonary arteries. The damage may be segmental or spread to the entire thoracic and abdominal aorta and its branches. Within the vasculitis of the large vessels of the Chapel Hill classification, Takayasu’s arteritis differs from giant cell arteritis, which affects older subjects. There is no other primary vasculitis of large vessels in children.

### 1.2 Epidemiology

Originally described in Japan, Takayasu’s arteritis is ubiquitous, but is observed with greater frequency in Asia. The annual incidence is estimated between 2 and 3 cases per million inhabitants, and in France between 1.2 and 2.6 cases/million/year. Due to its rarity and its chronic nature, the prevalence of Takayasu’s arteritis is difficult to assess. Estimated at 40 cases per million inhabitants in Japan, it is more like 4.7 per million people in the United Kingdom. In most cases, Takayasu’s arteritis affects young women. The third decade of life is the period when the incidence of occurrence is highest. The age of onset of the disease is between 20 and 40 years but remains difficult to assess. However, Takayasu’s arteritis mainly affects people under the age of 50. Women are the most affected, with a variable female-to-male ratio of 8/1 in Japan, 5.9/1 in Mexico, 4.8/1 in France and 1.2/1 in India.

In children, female predominance is probably not as clear as in adults. The median age of onset is about 10 years, although there are exceptional cases described before the age of 5 years. Ethnicity has not been specifically studied, the main series being Western—but it seems a predominance of populations of Asian origin is observed.

## 2 Goals of the national diagnostic and care protocol

### 2.1 Objectives

This PNDS can be used as a reference for the attending physician (a doctor indicated by the patient to the Primary Health Insurance Fund) in consultation with the specialist doctor, especially when establishing the care protocol jointly with the consulting doctor and the patient, in the case of a request for exemption from the copayment due to an off-list condition.

However, the PNDS cannot consider all the specific cases, all the comorbidities or complications, all the therapeutic peculiarities, all the hospital care protocols, etc. It cannot claim to be exhaustive regarding the possible management approaches, nor can it replace the individual responsibility of the physician vis-à-vis his patient. The protocol does, however, describe the reference treatment for a patient with Takayasu’s arteritis. It needs to be updated based on new validated data.

### 2.2 Work method

This PNDS was drawn up using the "Method of drawing up a national protocol for the diagnosis and care of rare diseases" published by the French National Health Authority (HAS) in 2012 (technical guide available on the HAS website: www.has-sante.fr). We identified all publications of interest in PubMed with the term "Takayasu" in the title in English or French until 2020. These recommendations are mostly based on retrospective studies, few prospective studies and experts opinions.

## 3 Diagnosis and initial assessment

### 3.1 Objectives

- To know how to recognize the symptoms suggestive of Takayasu’s arteritis early on.
- To be given the means to confirm Takayasu's arteritis and quickly rule out differential diagnoses.
- To look for and anticipate possible complications from Takayasu’s arteritis.

### 3.2 Professionals involved (and coordination terms)

The attending physician or outside pediatrician may be led to bring up the initial diagnosis of Takayasu’s arteritis, causing him to quickly refer his patient suspected of having Takayasu’s arteritis to a specialized center. Many other adult or child specialists may suspect Takayasu’s arteritis: cardiologists, radiologists, vascular physicians, internists, rheumatologists and ophthalmologists Confirmation of the diagnosis is ideally coordinated by a specialist physician with expertise in Takayasu’s arteritis, usually a vascular doctor or an internist.

### 3.3 Diagnosis

**Box 1.**

**Table:** 

*Key points (diagnosis)* The diagnosis of Takayasu’s arteritis is brought up in a subject (most often female) under the age of 50, in the presence of characteristic imaging* of the large arteries and in the absence of arguments for another vascular cause. The presence of clinical signs whose association is characteristic and/or inflammatory biological parameters may reinforce the diagnosis but may not exist and are not a prerequisite for making the diagnosis. *PET,, angio-CT, angio-MRI or a Doppler ultrasound.	

#### 3.3.1 Circumstances of discovery

The diagnosis may be made in a symptomatic patient under 50 years of age with general signs or ischemic symptoms with stress or high blood pressure. But it may also be a fortuitous discovery in the face of an absence of pulses or vascular murmurs on the clinical examination, or an arterial parietal thickening of the aorta or its branches, sometimes combined with arterial stenosis or aneurysms on the imaging tests. Finally, certain inflammatory pathologies are associated with Takayasu’s arteritis: inflammatory diseases of the digestive tract (Crohn's disease and ulcerative colitis), spondyloarthropathy and more rarely sarcoidosis. These may exist prior to Takayasu’s arteritis; it is therefore sometimes when following up on these pathologies that Takayasu’s arteritis is diagnosed.

**Box 2.**

**Table:** 

*Diagnostic circumstances* Takayasu’s disease should be considered if there are one or more of the following symptoms in a patient (particularly a woman) under the age of 50: *Clinical history*: Claudication of an upper limb Absence of a pulse of an upper limb Blood pressure asymmetry Renovascular hypertension Cervical murmur or subclavian murmur Pain along a vascular path, especially carotid *Imaging*: Aortitis Ostial coronaritis (proximal coronary stenosis) Parietal thickening of the supra-aortic trunks	

#### 3.3.2 Clinical manifestations

The circumstances for discovering the disease can be very different. In some patients, the diagnosis is made during the so-called "pre-occlusive" phase or the "systemic" phase. This phase combines general non-specific signs with fever, arthralgia, myalgia, skin signs (erythema nodosum, pyoderma gangrenosum), carotidodynia, and sometimes episcleritis.

In practice, the systemic phase often goes unnoticed, is absent or is found only retrospectively by questioning, or finally, concomitant with the occlusive phase.

The diagnosis is most often made during the "vascular phase", which is the result of arterial lesions (stenosis, obliterations, aneurysms) seated on the aortic arch, on the thoraco-abdominal aorta or their branches. It is often present from the onset when the patient comes in for symptoms related to stenosis or arterial occlusion. In this case, the way it is revealed depends on the area impaired.

##### 3.3.2.1 Involvement of the aortic arch and supra-aortic trunks

Axillo-subclavian involvement (typically post-vertebral) can cause upper limb claudication but is often asymptomatic, revealed by blood pressure asymmetry, an absence of pulses, a subclavicular murmur or Raynaud’s phenomenon. The presence of a vertebral-subclavian steal may be found.

Carotid artery involvement, which occurs in between 10 and 30% of the cases upon diagnosis, can be manifested as neck pain along the path of the carotid arteries, called carotidodynia. The absence of a carotid pulse or murmur upon auscultation may be found during the clinical examination.

Symptoms resulting from these arterial impairments are most often non-focal: dizziness, lipothymia, syncope, temporary blurred or dark vision in both eyes. They are the result of a transient low cerebral flow, occurring especially when getting up. Headaches are common, whether or not they meet the criteria of a migraine without aura; in particular, they could be secondary to a compensating high blood flow in the outer carotid area. Brain infarctions are rare, even when several arteries to the brain are affected. They can be the result of either arterial stenosis from Takayasu’s disease, or an indirect etiology such as atrial fibrillation with hypertensive heart disease, a lacuna or deep hemorrhage related to high blood pressure, or secondary atheromatous stenosis, and warrant a complete etiological investigation.

Ischemic retinopathy secondary to a decrease in retinal systolic pressure may be seen, as well as hypertensive retinopathy. It may be revealed by transient black spots in one eye triggered by orthostatism or intense ambient brightness.

##### 3.3.2.2 Involvement of the thoraco-abdominal aorta and the renal arteries

Intermittent claudication of the lower limbs may reveal the coexistence of stenosis and dilations or an aneurysm of the thoracic aorta or the abdominal aorta, which are very suggestive of the disease, especially when the vascular wall is thickened. Damage to the digestive vessels, celiac trunk and mesenteric arteries is quite common, but the occurrence of mesenteric angina is rare. Finally, renal artery stenosis, which is common, may lead to renovascular hypertension.

##### 3.3.2.3 Involvement of the lung vasculature

Pulmonary artery involvement occurs in about 50% of the cases. Most often asymptomatic, pulmonary arterial manifestations may be varied: chest pain, cough, dyspnea, and more rarely hemoptysis or pulmonary arterial hypertension with right heart failure.

##### 3.3.2.4 Cardiac involvement

Clinical myocardial involvement is rare, but non-specific perfusion abnormalities without related coronary involvement are frequently observed on a thallium scintigraphy (84%), as are delayed enhancements after injection of gadolinium (26%) on the MRI.

Coronary involvement affects 5–15% of the patients, mainly related to ostial stenosis combined with aortitis and most often manifested as angina.

Valvular involvement affects 2 out of 5 patients, mainly due to aortic insufficiency related to dilation of the aortic ring and the ascending aorta. Aortic insufficiency is a factor for a poor prognosis and needs to be corrected if the leak is significant. However, aortic insufficiency should be reassessed after controlling the high blood pressure. Finally, in the case of aortic pseudo-coarctation by stenosis of the descending or abdominal thoracic aorta, it will be treated first before the indication of an aortic valve replacement.

Hypertrophic heart disease or even left heart failure with renovascular arterial hypertension may occur.

##### 3.3.2.5 Hypertension

It is very common in Takayasu’s arteritis. It can be due to various etiologies: renal arterial impairment, aortic pseudo-coarctation and parietal rigidity secondary to the vascular impairment, increase in pulse pressure in cases of aortic valve insufficiency. Blood pressure numbers are frequently underestimated in the case of bilateral subclavian impairment: in this case, it is essential to monitor the systolic blood pressure by measuring the ankles, and the same blood pressure objectives will be retained.

##### 3.3.2.6 Dermatological impairment

The dermatological manifestations often correlated with flare-ups of the disease are mainly pyoderma gangrenosum and erythema nodosum, which are found in about 10% of cases. Skin signs are non-specific and may also be related to inflammatory pathologies related to Takayasu’s arteritis: inflammatory diseases of the digestive tract (Crohn's disease and ulcerative rectocolitis), spondyloarthropathy and, more rarely, sarcoidosis.

##### 3.3.2.7 Pediatric clinical characteristics

It is predominant in females but this is less clear than in adults. Children have more aortic and kidney impairment, explaining the frequency of high blood pressure, estimated at around 75% (benchmark definition of the National Health Institute). Digestive complaints, headaches, malaise and fever are also common. Skin and eye impairment and adenopathies are rarely seen. The absence of pulses and claudication of a limb appear to be found less often than in adults. Conversely, joint and muscle pain, as well as weight loss, appear to be more noted than in adults.

#### 3.3.3 Biological signs

There is no specific diagnostic biological marker or specific antibody of Takayasu’s disease. Inflammatory syndrome is confirmed by an increase in C-reactive protein (CRP), fibrinogen, 2-globulin, haptoglobin, orosomucoid and/or the erythrocyte sedimentation rate (ESR). The inflammatory syndrome is erratic, and its absence does not necessarily signify the absence of activity of the disease. The search for antinuclear factors or rheumatoid factors is negative in the absence of associated collagen disease. The presence of anti-endothelial cell antibodies has been reported but is not specific. Pentraxin 3 has been described as a marker of disease activity. CRP belongs to the family of pentraxins, but is synthesized by the liver, unlike pentraxin-3, which is produced by immune cells and cells of the arterial wall. However, pentraxin 3 is not determined in the clinical routine.

In pediatric series, the parameters most often noted as being high are the ESR and then CRP, the use of which as a reflection of activity also remains uncertain. Chronic inflammation is more often combined with anemia and thrombocytosis in children than in adolescents.

#### 3.3.4 Imaging of the aorta and its branches (Fig. 1a, b)

**Fig. 1:**
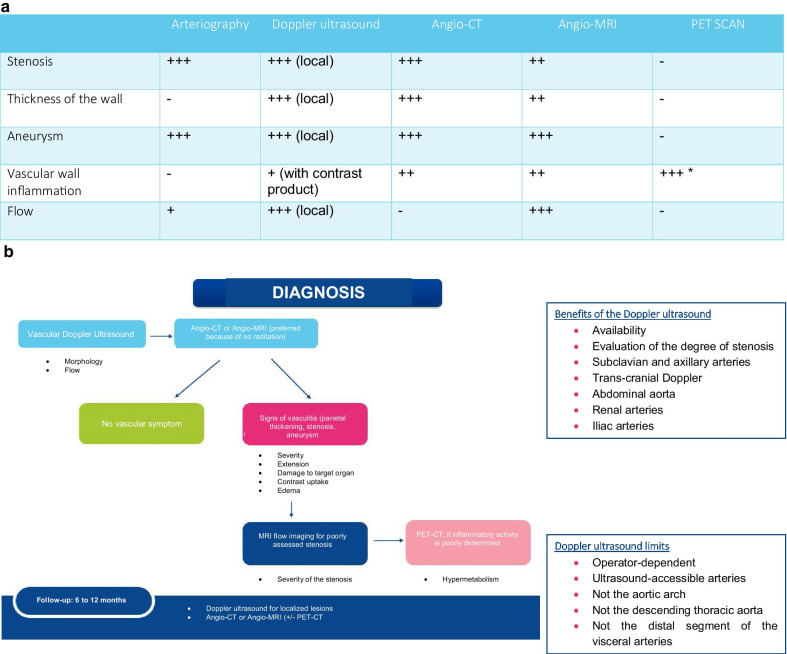
**a** Imaging during Takayasu’s arteritis. *More sensitive on the initial diagnosis. Angio-CT and angio-MRI of the thoracic aorta and abdominal aorta, and angio-CT and angio-MRI of the supraortic trunks (SAT). **b** Role of tests in the diagnosis and follow-up of Takayasu’s arteritis.

The purpose of the images of the aorta and its branches is:

1. The search for morphological impairment of the aorta and/or its branches suggestive of aortitis (arterial parietal thickening and remodeling, stenosis, aneurysm).
2. The detection of arterial parietal inflammatory activity.

Arterial imaging plays a major role in the positive diagnosis of the disease and the ongoing follow-up. The search for involvement of the aorta and its branches is carried out by the arterial Doppler ultrasound, angio-CT or angio-MRI. Whole-body arteriography, mentioned in the 1990 American College of Rheumatology classification criteria, is no longer used for diagnosing and following up on patients. A positron emission tomography or PET scan has been proposed to study arterial parietal inflammatory activity.

The vascular Doppler ultrasound shows a typical hypoechoic perivascular halo and measures the thickness of the carotid and axillary walls with good sensitivity. This type of imaging does not determine disease activity and is less sensitive than cross-sectional imaging (angio-CT, angio-MRI) for inflammatory lesions of the visceral arteries, in the absence of hemodynamic stenosis. Its use is also limited by the inaccessibility of certain vessels such as the thoracic aorta and pulmonary arteries. Evaluation of the inflammatory activity of Takayasu’s arteritis by the use of ultrasound contrast product has been illustrated but has yet to be validated.

An angio-CT and angio-MRI are worthwhile when they show parietal thickening or when they allow viewing of arterial stenosis and aneurysms. The advantage of the angio-CT is an excellent isotropic spatial resolution covering the entire arterial tree and great accessibility. The angio-CT will be synchronized to the heart rate with arterial phase and delayed phase, making it possible to evaluate the parietal contrast uptake. Aortitis is characterized by aortic, thoracic or abdominal parietal thickening, circumferential and regular in appearance, greater than or equal to 2–3 mm, sometimes being enhanced after injection. The advantage of the angio-MRI (including magnetic resonance angiography and dedicated MRI sequences) is the absence of any radiation or iodized contrast product, as well as the possibility, in addition to the morphological study, of detecting signs of vascular inflammation such as parietal edema using T1-weighted imaging with and without an injection of gadolinium as well as in T2 imaging with fat saturation. Its non-radiating nature makes the MRI particularly appropriate for following up on young patients. However, although the role of this technique in the diagnostic assessment of vascular lesions is established, the assessment of the inflammatory activity of Takayasu’s arteritis has been demonstrated but has yet to be validated. A PET with FDG is a technique that aims at a more direct approach to the degree of vascular inflammation by estimating increased consumption of radioactive glucose in the inflammatory cell infiltrate of the arterial wall. The most commonly used setting for a PET scan with FDG is a visual analysis, comparing the degree of aortic uptake to that of the liver, which categorizes the examination into three grades (grade 1: lower uptake than the liver; grade 2: aortic uptake equal to that of the liver; grade 3: aortic uptake greater than that of the liver). Vascular hypermetabolism on the PET does not necessarily determine an active disease. The lack of uptake does not rule out a progression of the disease. This examination is part of a group of arguments to assess the activity of the disease but is not sufficient by itself to modify the therapeutic approach.

The sensitivity and specificity of these imaging techniques for the study of inflammatory activity remain to be determined by comparative studies on larger cohorts.

In the pediatric forms, no comparative data are available to date; some pediatric studies highlight the value of the angio-MRI (less radiation than the angio-CT) and the Doppler ultrasound for renal and carotid arteries—the radiation issue being major as to the oncogenic risk. Abdominal impairment more or less related to other topographies is the most common.

##### 3.3.4.1 Anatomopathological examination

The histological examination cannot be systematic, due to the site of the vascular impairment, but it can be performed in case of a procedure required for arterial revascularization. It involves a predominantly medial-adventitial giant-cell panarteritis. In the acute phase, the predominant abnormalities of the adventitia are an inflammatory infiltrate, replaced by fibrosing lesions and arterial calcifications in the chronic phase, active lesions and fibrosis possibly occurring simultaneously.

#### 3.3.5 Diagnostic principles and classification criteria

The diagnosis of Takayasu’s arteritis is made on a group of clinical, biological, radiological and sometimes histological elements.

Classification criteria exist, the most commonly used being the American College of Rheumatology (ACR) criteria and the Ishikawa criteria modified by Sharma. The ACR criteria take into account primarily arterial involvement and the characteristic aspect on imaging, ruling out other possible causes of stenosis and/or thrombosis. The ACR criteria have a sensitivity of 90.5% and a specificity of 97.8% but have not been reviewed in view of new vascular imaging techniques. (Table 1)

**Table 1:** ACR Criteria (1990) for the diagnosis of Takayasu’s arteritis

Age of onset ≤ 40 years	
Claudication of the extremities: discomfort or muscle fatigue upon effort of at least one extremity, especially the upper limbs	
Decrease of at least one brachial pulse Asymmetry of at least 10 mm Hg of the	
humeral systolic pressure	
Murmur upon auscultation on a subclavian artery or on the abdominal aorta	
Angiographic abnormalities (angio-CT and/or angio-MRI are the imaging modalities currently used): narrowing or occlusion on the aorta, its branches or the proximal arteries of the limbs, segmentalor focal, not related to the atherosclerosis or fibromuscular dysplasia	

Ishikawa's criteria of 1988 modified by Sharma et al. in 1996 incorporate vascular and systemic signs, also considering the presence of stenosis or vascular occlusion on the arteriography. (Table 2)

**Table 2:** Ishikawa criteria (1988) modified by Sharma (1996) for the diagnosis of Takayasu’s arteritis

*Major criteria*	
1. Stenosis or occlusion of the middle portion of the left subclavian artery on the arteriography	
2. Stenosis or occlusion of the middle portion of the right subclavian artery on the arteriography	
3. Characteristic symptoms lasting at least one month: claudication, absence of a pulse or blood pressure asymmetry, fever, cervicalgia, amaurosis, visual disorders, syncope, dyspnea, palpitations.	
*Minor criteria*	
1. SR > 20 mm/h	
2. Sensitivity of carotid arteries to palpation	
3. High blood pressure: humeral pressure—140/90 mmHg, or popliteal pressure—160/90 mmHg	
4. Aortic insufficiency or annuloaortic ectasia	
5. Pulmonary arterial impairment	
6. Stenosis or occlusion of the middle portion of the left carotid artery on the arteriography	
7. Stenosis or occlusion of the distal third of the brachiocephalic artery trunk on the arteriography	
8. Impairment of the descending thoracic aorta on the arteriography	
9. Impairment of the abdominal aorta on the arteriography	
10. Coronary heart injury before the age of 30, in the absence of dyslipidemia or diabetes	
A diagnosis of Takayasu’s arteritis is highly likely if: ≥ 2 major criteria or 1 major criterion and ≥ 2 minor criteria or ≥ 4 minor criteria	

In general, a diagnosis of Takayasu’s arteritis is made in a subject under 50 years of age, if there is a characteristic radiological impairment of the large caliber arteries and there are no arguments for another vascular cause. The presence of characteristic clinical signs and/or inflammatory biological parameters may reinforce the diagnosis, but they may be absent and are not a prerequisite for making the diagnosis.

In children, European consensus criteria validated on an international cohort were drawn up and published in 2010—PRINTO/EULAR/PreS—with a sensitivity of 100%, a specificity of 99.9%. The diagnosis of pediatric Takayasu’s arteritis is made for a case beginning at 18 years of age and less with:

- An angiographic abnormality (angio-CT or angio-MRI or arteriography) of the aorta or its main branches or pulmonary branches; type of aneurysm/dilation, stenosis, occlusion, parietal, segmental or focal thickening; with no argument for fibromuscular dysplasia or any other vascular cause;
- + 1/5 criteria including;
- Absence, lowering or asymmetry of the arterial pulse; claudication of a member upon effort;
- Blood pressure differential > 10 mm Hg;
- Vascular murmur of the large vessels, audible or palpable;
- HIGH blood pressure—95th percentile for size;
- SR > 20 mm or CRP > the laboratory’s range.

### 3.4 Differential diagnoses

For typical forms of Takayasu’s arteritis, the question of an alternative diagnosis hardly comes up. In other cases, differential diagnoses of Takayasu’s arteritis are as numerous as the presentation is

A differential diagnosis with giant cell arteritis (formerly known as Horton's disease) may be difficult, especially if inflammatory arterial lesions are found in a person over 50 years of age. Age is not a sufficient discriminating factor, because arterial lesions related to Takayasu’s arteritis may go unnoticed in a young patient and be discovered if the disease progresses, there is a systematic assessment or there are consequences of arterial lesions several years after the onset of the disease. Signs of cephalic arteritis (headaches, scalp abnormalities) and characteristic abnormalities present upon performance of a temporal artery biopsy are more discriminating elements suggestive of giant cell arteritis. In contrast to TA, subclavian and axillary diseases seem to cluster symmetrically in GCA. Compared with GCA, the carotid, renal and mesenteric arteries are more frequently affected in TA.

Other etiologies of aortitis must be systematically brought up and discarded. An infectious origin (mainly related to tuberculosis, syphilis, or in better defined clinical pictures staphylococcus, streptococcus and salmonella) will be brought up depending on the clinical context and with an appropriate biological assessment. Other inflammatory and/or autoimmune pathologies, such as Behcet's disease, Cogan’s syndrome, relapsing polychondritis, rheumatoid arthritis or granulomatosis with polyangiitis, will be brought up if there are characteristic clinical signs and/or specific autoimmune markers. Other non-inflammatory vascular pathologies such as atheroma, genetic vascular diseases (Marfan syndrome and related syndromes, Loeys-Dietz syndrome, Williams-Beuren syndrome, vascular Ehlers-Danlos syndrome), post-radiation arteritis and fibromuscular dysplasia, will be eliminated on personal and family histories and the patient’s clinical and radiological characteristics. Periaortitis (Erdheim Chester disease, IgG4 disease, perianeurysmal retroperitoneal fibrosis ...) can also be suggested.

In pediatrics, in addition to the diagnoses cited above, there may be diagnostic confusion in the types of Takayasu’s arteritis extended to the roots of the middle vessels with Kawasaki disease (coronary impairment) and polyarteritis nodosa (mesenteric and kidney impairment).

### 3.5 Assessment of the disease activity

There are no reliable criteria for the progression of Takayasu’s arteritis, and there are no specific biological markers, such as the presence of specific antibodies. The usual autoimmune assessment is negative. The biological signs are related to an inflammatory syndrome, which results in an increase in the inflammatory parameters: ESR, CRP, fibrinogen, orosomucoid, haptoglobin. An inflammatory syndrome, however, remains an imperfect reflection of the activity of the underlying disease, an active disease being possible in the absence of an inflammatory syndrome in approximately 30% of the patients. Conversely, an inflammatory syndrome can be observed in about 30% of the patients without angiographic progression. Other biological markers have been studied, such as the levels of various cytokines, higher levels of RANTES, TNF-α and IL-6 being detected in cases of active disease, and other markers, such as pentraxin 3 or BAFF, are also high in cases of active disease, but these markers are not routinely achievable.

Some criteria for Takayasu’s arteritis activity have been defined based on the prospective follow-up of a series of 60 patients from the National Institute of Health (NIH). They are based on the recent onset or aggravation of at least 2 of the following 4 criteria:

- Signs of ischemia or vascular inflammation.
- Systemic symptoms not attributable to other events.
- Angiographic abnormalities.
- An increase in inflammation markers (ESR, CRP, fibrinogen, orosomucoid, haptoglobin).

In this study, 88% of the patients with a clinically active disease saw the onset of new arterial impairment. More recently, a Turkish team tried to validate an activity score of Takayasu’s arteritis (Disease Extent Index-Takayasu). The Indian teams use another score, the ITAK, the concordance of which seems very close to the NIH activity score. Some tests to follow up on Takayasu’s arteritis by positron emission tomography have been performed, but the sensitivity and specificity of this imaging are not yet known. MRI was also proposed because of its ability to identify parietal edema, but anatomopathological and clinical-biological correlations have not yet been established. The activity criteria defined by the NIH are currently the most used in practice to determine and monitor the activity of the disease.

### 3.6 Progression and prognosis

The progression is quite variable. Despite current treatments, about 50% of the patients relapse or develop a vascular complication within 10 years of the diagnosis of Takayasu's disease. Vascular lesions may progress in a completely silent way. The assessment of the activity of Takayasu’s arteritis meets clinical, biological and imaging criteria. Remission is defined as the absence of new symptoms, the absence of an inflammatory syndrome and the absence of any radiological modification. Sustained remission should be achieved with an objective of prednisone of < 0.1 mg/kg/d. In a recent French study, after a median follow-up of 6.1 years, relapses were observed in 43% of the cases, vascular complications in 38% of the cases and deaths in 5% of the cases. The rates of event-free survival at 5 and 10 years, of relapse-free survival and survival without complications were 48.2% and 36.4%, 58.6% and 47.7% and 69.9% and 53.7%, respectively. Being male, high CRP and carotidynia were associated with the risk of a relapse. The gradual progression of the disease, thoracic aorta impairment and retinopathy were associated with the risk of vascular complications.

The prognosis for survival of Takayasu’s arteritis is generally good. However, Takayasu’s arteritis is associated with increased mortality. Survival at 15 years is 85% and at 20 years 75% in previous studies dating back to the 1980’s. The standardized mortality rate (SMR) was 3.0 compared to the general population of the same age. Early identification of patients with poor prognosis factors could help prevent these deaths.

The prognosis after a stroke has not been specifically studied, but strokes are regularly cited as a cause of death in published series. Cardiomyopathy due to aortic insufficiency, hypertension or coronary artery disease or combinations is an important cause of morbi-mortality in these patients.

In early pediatric Takayasu’s arteritis, there is little evidence for determining a 5-year mortality rate, which seems to fluctuate between 0 and 40%. It is that much higher if the onset is early (5 years) and the disease is active. Gender, ethnicity, delay in the diagnosis and nature of the impairments do not seem to impact the prognosis. The rate of remission seems to improve with the use of biotherapies (a study reporting remission in 2 years in 80% of patients). Adult TA may have a better prognosis than children in a Brazilian study. Typical complications related to high blood pressure are common.

## 4 Therapeutic management

**Box 3.**

**Table:** 

*Key points (treatment)* The treatment of the conventional approach is prednisone at a dose of 0.5 to 1 mg/kg/d (maximum dosage of 70 mg/day) in the inflammatory phase. The goal is to control the activity of the disease while gradually reducing the doses of prednisone. When we reach 5 to 10 mg/d of prednisone, a frequently adopted approach is a reduction of prednisone by 1 mg per month, and the final halt in the corticosteroid therapy must be systematically attempted. Experts suggest putting immunosuppressants (i.e.methotrexate) systematically on as the first line for the purpose of reducing the use of steroids. The immunosuppressants for which there is the most data are methotrexate. Biotherapies have also been shown to be effective in these situations, including anti-TNF-alpha (infliximab, adalimumab, etanercept) and anti-IL6 (tocilizumab).	

### 4.1 Objectives

To date, there has been no therapeutic consensus concerning Takayasu’s arteritis. Most of the data comes from retrospective series and small cohorts of patients. The treatment of Takayasu’s arteritis is based on the medical treatment, which aims to treat the inflammatory aspect of the disease, and in some cases on revascularization by angioplasty or surgery.

### 4.2 Professionals involved (and coordination arrangements)

The treatment is ideally coordinated by the specialist doctor with expertise in Takayasu’s arteritis, usually a vascular doctor or an internist. Other professionals may be involved, such as cardiologists or nephrologists in cases of renovascular hypertension, gastroenterologists in case of associated inflammatory bowel disease; rheumatologists for associated spondyloarthropathy or corticosteroid-induced fractures from osteoporosis, ophthalmologists for specific eye impairment, pulmonologists for PAH, radiologists and vascular surgeons in the event a revascularization procedure is considered.

### 4.3 Search for contraindications to the treatment

The treatment of Takayasu’s arteritis involves specific drugs for curative purposes and also takes into account the treatments (preventive or curative) for complications.

**Box 4.**

**Table:** 

*Therapeutic management* *Objectives*: Limit vascular complications and revascularizations and control vascular inflammation Avoid relapses Limit the side effects of the corticosteroid therapy Controlthe frequently associated high blood pressure *Measures to be implemented in all cases*: Management of cardiovascular risk factors, particularly high blood pressure Anti-platelet antiaggregant drug (in the absence of a contraindication) Statin: often used in this context in primary prevention in spite of a lack of data in the literature Updated vaccine schedule—pneumococcal vaccination and seasonal flu shot *Specific treatment*: Only in the inflammatory phase and/or in case of progression Corticosteroid therapy: prednisone 0.5 to 1 mg/kg/day as a first-line therapy	

#### 4.3.1 Means: available drugs

In the inflammatory phase of the disease, oral corticosteroid therapy is the specific reference treatment for Takayasu’s arteritis. The drug usually prescribed is prednisone. All other specific treatments for Takayasu’s arteritis are prescribed in combination with the corticosteroid therapy: immunosuppressants (methotrexate, azathioprine, mycophenolate mofetil) and targeted biotherapies (anti-TNF, anti-IL6). Experts suggest putting methotrexate systematically on as the first line for the purpose of reducing the use of steroids.

Adjuvant treatments, such as methotrexate or targeted biotherapies (anti-TNF, anti-IL6), are the main immunosuppressants used.

#### 4.3.2 Therapeutic principles and means

##### 4.3.2.1 Corticosteroid therapy

Corticosteroid therapy is recommended in active forms of the disease. The dosage of prednisone generally prescribed in the initial treatment ranges from 0.5 (mild forms with limited arterial impairment) to 0.7–1 mg/kg/day (without exceeding 70 mg/day) in more severe forms (vital organ damage, multiple and/or progressive arterial lesions, renovascular hypertension, vascular kidney failure, coronaritis, ischemia of a limb, cerebral infarction related to arteritis of the supra-aortic trunks, symptomatic intestinal ischemia, aortic insufficiency). The administration of a corticosteroid bolus (methylprednisolone) is not indicated except in severe life-threatening forms.

**Fig. 2:**
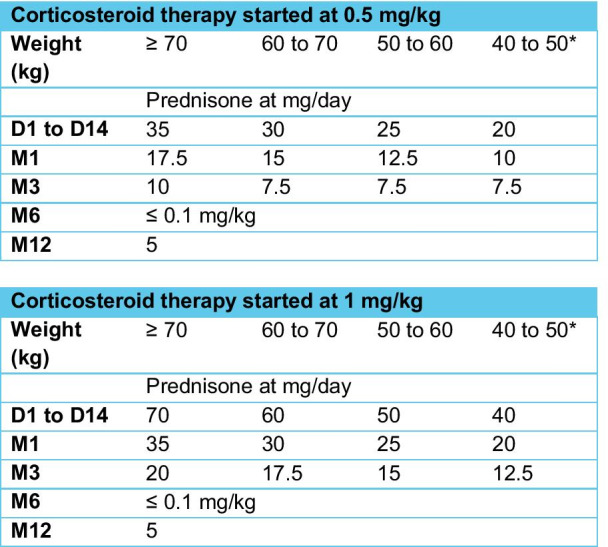
General regime of corticosteroid therapy approach (started at 0.5 or 1 mg/kg/day) for a patient with uncomplicated Takayasu’s arteritis with no relapse. *For low-weight pediatric patients, refer to the pediatric reference or competence centers.

The initial dose is usually maintained for 2–4 weeks. The reduction phase can begin as soon as the clinical signs and biological inflammatory syndrome have significantly improved. During this reduction phase, the suggested objectives of the prednisone dose to be reached are 15–20 mg/day in the 3rd month, and ≤ 0.1 mg/kg/day in the 6th month from the start of treatment (Fig. 2).

When we reach 5 mg/d of prednisone 5 mg/day a frequently adopted approach is a reduction of prednisone by 1 mg per month and/or to perform a synacthen test., and the final halt in the corticosteroid therapy must be systematically attempted.

The purpose of cautiously decreasing prednisone is twofold: on the one hand, to identify a possible effective minimum dose of corticosteroids to keep Takayasu’s arteritis in remission and, on the other hand, to allow the adrenal secretion to recover so as to ensure adequate secretion of endogenous cortisone. Usually, an attempt to discontinue corticosteroid treatment is considered after 24 months of quiescent disease.

Corticosteroid therapy is effective when the diagnosis of the disease is made early, in the pre-occlusive phase. Corticosteroid therapy alone can achieve remission in 25–50% of the cases. Relapses during the reduction of corticosteroids are described in 30–40% of the cases. In the NIH prospective series bearing on 60 patients, 48 had signs of disease activity and received corticosteroid therapy (prednisone 1 mg/kg/day). Out of these 48 patients, 25 (52%) went into remission. Sixteen (64%) patients relapsed and were treated again with corticosteroids, with a favorable response in half of the cases. Long-term corticosteroid therapy can be harmful in this pathology especially because of the cardiovascular complications it can cause. According to the series, 20–50% of patients develop side effects related to corticosteroid therapy, including cataracts, peripheral edema, myopathy, fracture, infection and diabetes.

In pediatrics, the oral dose of prednisone is similar to that described for adults. The indication of boluses of methylprednisolone—more readily at a dose of 15 mg/kg over 3 days—is broader than in adults, to be discussed for those forms active at diagnosis and/or which risk becoming so. We especially emphasize the adverse impact of long-term steroids on growth; this has been confirmed elsewhere in an English series. The psychological impact of secondary Cushing’s syndrome may lead to staying out of school and poor adherence to the treatments. Osteoporosis and the risk of aseptic necrosis of the femoral head also exist in children. These issues, combined with the increased medium- and long-term risk of complications or even death from the disease, are all reasons for discussing from the outset a treatment that attempts to limit the use of corticosteroids.

The pediatric dose is usually 1–2 mg/kg/day in the initial treatment, to be discussed on a case-by-case basis, depending on the child's weight and the context.

##### 4.3.2.2 Methotrexate

**Fig. 3:**
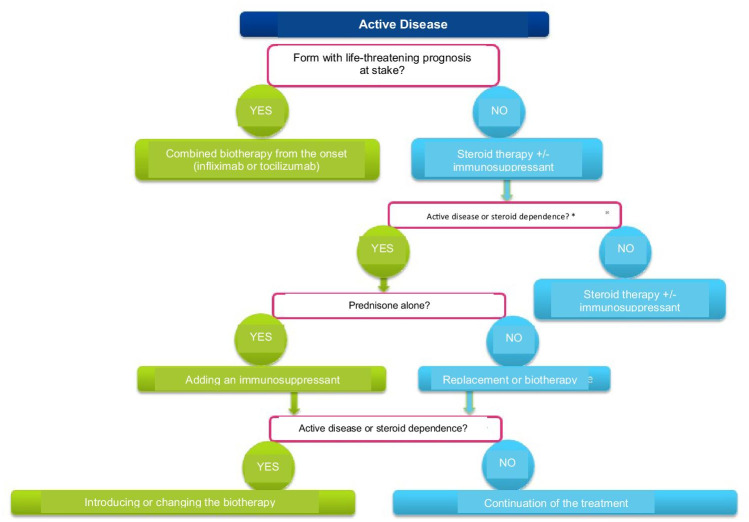
Specific treatment procedure for Takayasu’s arteritis

Most experts recommended the use of methotrexate as the first line for the purpose of reducing the use of steroids (Fig. 3). The use of methotrexate in this indication appears to be worthwhile, but this treatment has only been evaluated in open studies and on low numbers of subjects. Methotrexate is generally prescribed at doses of 0.3 mg/kg/week. The addition of methotrexate to corticosteroids makes remission possible in 50–80% of corticosteroid resistance or dependence cases. In a pilot study of 16 patients with cortico-resistant Takayasu’s arteritis, 13 out of 16 (81%) went into remission. Relapses occurred in 44% of the cases (7 out of 13 patients). After an average follow-up of 18 months, 50% of the patients remained in prolonged remission. The period for continuing the immunosuppressant treatment is not clearly defined in Takayasu’s arteritis. Methotrexate is teratogenic (Teratogenic Agent Reference Center (www.lecrat.fr) (neural tube closure abnormalities), and therefore contra-indicated during pregnancy or while breastfeeding.

In children, methotrexate is the most commonly used corticosteroid-reducing treatment—its use is published in about 50 patients, with efficacy that cannot be directly assessed. The published doses range from 10 to 15 mg/m^2^/week—usually no more than 20 mg/week for a total period of 18 to 24 months. The use of the subcutaneous form helps to limit digestive side effects like nausea and optimizes adherence—checked by a nurse or the parent performing the injection. The tolerance seems good; only one study emphasizes the occurrence of 4 cases of candidiasis, including one person hospitalized, in children otherwise on long-term oral prednisone.

##### 4.3.2.3 Azathioprine and purinethol

Azathioprine (2–3 mg/kg/day, maximum 150 mg/day) in combination with corticosteroids in 65 patients, making possible a clinical remission, as well as an absence of arterial progression for a 12-month follow-up period. By contrast, there was no improvement in the pre-existing radiological lesions when taking azathioprine. In the case of digestive intolerance of azathioprine, purinethol (1–2 mg/kg/day) can be tried.

In children, azathioprine is also used, to a lesser extent than methotrexate, so as to reduce the use of corticosteroids. It is used at a dose of 2 mg/kg/day.

##### 4.3.2.4 Mycophenolate mofetil

Among 10 patients treated with mycophenolate mofetil (2 g/day), clinical remission and a reduction in the use of corticosteroids were obtained in all the patients. In another study, 21 patients treated with mycophenolate mofetil had a clinical improvement and a reduction in the use of corticosteroids for a median follow-up period of 9.6 months.

The use of cyclophosphamide reported in older publications is no longer routinely used with the advent of biotherapies.

##### 4.3.2.5 Anti-TNF alpha

The value of biotherapies has been evaluated primarily in retrospective open studies in patients resistant to conventional immunosuppressants or corticosteroid-dependent.

TNF-α antagonists, mainly infliximab, have been evaluated in several open series. Clinical response rates (complete or partial) of about 80% and the steroid-sparing effect in these patients, most often having had several lines of immunosuppressants, seem to indicate that these agents are effective.

These results are corroborated by 5 pediatric series, one of which reported a benefit from the use of biotherapies in terms of remission without a relapse when treated for 2 years. The one most used is still infliximab, with about 20 published cases (described dose of 5–6 mg/kg in variable regimens at weeks 0–2–4–8, then every 8 weeks or weeks 0–2–4 and then every 4 weeks), with a 66% response. The other anti-TNFα less used is adalimumab (described dose of 40 mg/2 weeks in people > 30 kg or 24 mg/m^2^ for people < 30 kg). Their tolerance seems good—which often makes them preferred over cyclophosphamide. It may also be noted that the indication of anti-TNFα’s is more appropriate if spondyloarthritis or an inflammatory bowel disease is combined with arteritis.

The experience gained in the severe forms with biological treatments mainly involves the IV route. A preliminary experiment exists with some biological treatments via the subcutaneous route.

##### 4.3.2.6 Tocilizumab

Several retrospective open series have shown the effectiveness of tocilizumab, an anti-IL6R, in terms of clinical-biological remission and a steroid-sparing effect.

Comparison of TNFα antagonists to tocilizumab showed similar results in terms of clinical responses (61% and 70% at 6 months), with a similar frequency of vascular complications during follow-up, as well as survival at 3 years with no relapse, of 91% and 85.7%, respectively. During the follow-up, 21% of the side effects were noted in 49 treated patients, half of whom required discontinuation of the biotherapy. The frequency of complications was no different between TNFα antagonists and tocilizumab. In the same study, a comparison to conventional immunosuppressants appeared to favor biotherapies (TNFα antagonists and tocilizumab) in terms of survival with no relapse and no events; subject to the biases related to the retrospective analysis. It should be noted, however, that the radiological assessment, in particular, the effect of biotherapies on existing lesions and the emergence of new arterial locations, is usually not thoroughly analyzed. A randomized trial evaluating the efficacy of tocilizumab versus placebo in refractory Takayasu’s disease was recently published by a Japanese team (TAKT study). The time until the relapse was the main assessment criterion, and 18 patients were included in each treatment arm. The results of this study suggest a beneficial effect in favor of tocilizumab, although the main primary endpoint was not achieved in an intention-to-treat analysis.

About 10 reported cases of children treated effectively with tocilizumab with a good tolerance profile, make this treatment a second or third line to consider in the refractory forms. This was described at a dose of 8 mg/kg/ for 2–4 weeks (knowing that in children under 30 kg, the usual doses of tocilizumab can go up 10–12 mg/kg every 2–4 weeks).

In severe forms of TA most reports with biological treatments mainly involves the IV route but the SC route can be administered as in other diseases.

#### 4.3.3 Treatment of relapses and high-level corticosteroid dependent forms

Complete withdrawal from the corticosteroid therapy is not always easily achieved. A progressive clinical-biological relapse of Takayasu’s arteritis previously in remission by the treatment occurs in at least 40% of the cases.

In principle, relapses or recurrences are diagnosed based on the reappearance or concomitant aggravation of the clinical and/or radiological symptoms of Takayasu’s arteritis and the biological inflammatory syndrome.

The main issue is knowing to whom and when to prescribe the adjuvant treatment.

### 4.4 Related treatments, education on the therapy, lifestyle changes (on a case-by-case basis) and other treatments

#### 4.4.1 Vascular treatments

The risk of cardiovascular events should be assessed individually, and risk factors should be checked. In this way, a SCORE (www.heartscore.org) type of scale can help integrate all the risk factors (age, gender, body mass index, waist and hip circumference, use of tobacco, cholesterol, blood pressure, diabetes, physical activity) and provide a better assessment of the overall cardiovascular risk. This stratification results in lifestyle, psychosocial and drug adaptations based on the goals to be achieved. Cardiovascular screening tests are to be discussed on a case-by-case basis with a cardiovascular disease specialist in a comprehensive stratification strategy of the cardiovascular risk.

##### 4.4.2 Platelet antiaggregants and anticoagulants

The rationale for prescribing an antiaggregant treatment for patients diagnosed with Takayasu’s arteritis is weak.

In this context of uncertainty, it seems reasonable to restrict the prescription of an antiaggregant dose of aspirin (75–300 mg/day) to Takayasu’s arteritis with severe stenosis impairment. In children, the dose of aspirin is 3–5 mg/kg (no more than 75 mg/day); its usage varies between 30–60%.

Routinely prescribing an anticoagulant therapy is not recommended.

##### 4.4.2.1 Statins

The preventive impact of statins on the occurrence of cardiovascular complications has not been specifically demonstrated for Takayasu’s arteritis.

There is a complex interaction between inflammatory diseases and the development of atherosclerosis. Specific features related to inflammation and the nature of inflammatory disease lead to early atherosclerosis and an increased risk of atherosclerotic cardiovascular disease. Thus, it has been suggested that the cardiovascular risk determined based on conventional risk factors present in an individual be multiplied by 1.5. However, the presence of an inflammatory disease is not in itself a sufficient indication to routinely prescribe a statin. There are also no specific LDL-cholesterol targets in the case of inflammatory disease or targets lower than those recommended for individuals without inflammatory disease. The targets are as described below according to the ESC 2019 guidelines.

For patients with a low CV risk (<1% cardiovascular mortality at 10 years (SCORE scale)), the goal of LDL-cholesterol is to be less than 3 mmol/L (or 1.16 g/L), with dietary intervention before starting a treatment for LDL-c between 3 and 4.9 mmol/L, and the immediate introduction of a statin in addition to dietary intervention for all LDL-cholesterol > 4.9 mmol/L (or 1.9 g/L).

For patients with a moderate CV risk (1–5% cardiovascular mortality at 10 years), the goal of LDL-cholesterol is to be less than 2.6 mmol/L (or 1 g/L).

For patients with a high CV risk (5–10% cardiovascular mortality at 10 years), the goal of LDL-cholesterol is to be less than 1.8 mmol/L (or 0.7 g/L).

For patients with a very high CV risk (≥10% cardiovascular mortality at 10 years), the goal of LDL-cholesterol is to be less than 1.4 mmol/L (or 0.55 g/L). It is also the target recommended for all patients on secondary cardiovascular prevention and any patient with severe kidney failure (glomerular filtration rate less than 30 mL/min/1.73m^2^).

The hypolipidemics to be prescribed are first-line statins with increased doses in order to reach the target, and/or the addition of ezetimibe as the second line.

##### 4.4.2.2 Other vascular protection measures

In the case of structural damage to the aorta, the arterial pressure should be monitored according to the current guidelines for aortic aneurysms. Smoking is a risk factor for complications of aortitis, and all patients must stop smoking. The data currently available are not strong enough to recommend routinely prescribing an angiotensin-converting enzyme inhibitor (or an angiotensin II receptor antagonist) to patients newly diagnosed with Takayasu’s arteritis.

However, close attention should be paid to screen for high blood pressure and monitor high blood pressure that is being treated. The guidelines for managing high blood pressure do not change within the context of managing Takayasu’s arteritis. The necessary means should be ambulatory blood pressure measurements (ABPM) or self-measurements, and not just clinical measurements in a doctor’s office. Finally, blood pressure measurements at the ankles are often necessary both in screening and in follow-up due to frequent damage to the subclavian arteries which will lower the blood pressure numbers measured at the humerus. According to the ESH/ESC 2018 guidelines, high blood pressure occurs when blood pressure values are greater than 140 mmHg of systolic pressure or 90 mmHg of diastolic pressure.

The pressure targets for patients under 65 years of age are between 120 and 129 mmHg for systolic pressure and less than 80 mmHg for diastolic pressure. The pressure targets for patients 65 years of age and older are between 130 and 139 mmHg for systolic pressure and less than 80 mmHg for diastolic pressure.

A combination of two anti-hypertensive treatments is to be preferred when a treatment for high blood pressure is started. Combined treatments to be taken at one time are to be preferred. The combination of ACE inhibitors or ARA2 (angiotensin receptor antagonists 2) with a calcium channel blocker or a thiazide diuretic are to be preferred after ruling out renal artery stenosis.

The treatment of high blood pressure in children should target the size-appropriate standards published by the NIH (BP—95th percentile).

(http://pediatrics.aappublications.org/content/pediatrics/140/3/e20171904.full.pdf).

#### 4.4.3 Prevention of cortisonic osteoporosis

The importance of preventing cortisonic osteoporosis is often underestimated. Prevention aims to limit the risk of fracture in patients taking long-term corticosteroid therapy (≥7.5 mg/day of a prednisone equivalent for 3 months). Some French guidelines were issued in 2014; they consider the case of non-menopausal women and men under 50 years of age, the main population concerned with Takayasu’s arteritis but for whom the approach to be taken in the prevention of cortisonic osteoporosis is less obvious than for an older population.

##### 4.4.3.1 Initial assessment of the fracture risk

- Bone densitometry (DXA) is recommended for patients starting prolonged corticosteroid therapy (≥7.5 mg/day of a prednisone equivalent for 3 months).
- Sub-clinical fractures should be screened by X-rays of the spine in case of a loss of height of ≥ 4 cm compared to the height at 20 years of age, loss of height of ≥ 2 cm during follow-up, if there is spinal pain, or in children.
- The use of the FRAX® score (determination of the fracture risk within 10 years) is not validated in non-menopausal women or men under 50 years of age.
- The measurement of bone turnover markers is of no importance.

##### 4.4.3.2 General measures

- Prescribe the minimum effective corticosteroid dose.
- Encourage adequate daily calcium intake, preferably through diet; routinely prescribing calcium is not recommended.
- Measure the level of 25-OH vitamin D and keep it above 30 ng/mL.
- Encourage physical activity.
- Smoking must be discontinued.
- Avoid excessive alcohol consumption.

##### 4.4.3.3 Specific treatment for osteoporosis

In non-menopausal women and men under 50, the risk of a fracture is less, and the use of specific treatments becomes difficult due to the lack of data from the literature on the efficacy of these treatments in this population and on the risk associated with the use of bisphosphonates in women in the event of a subsequent pregnancy.

This prescription should only be considered if fragility fractures (including subclinical vertebral fractures) are found.

In all cases, the treatment decision should be made after an individual assessment (underlying pathology, dose and duration of the corticosteroid therapy, results of the DXA) and should be subject to a specialized consultation.

In this population, bisphosphonates are used outside of the marketing authorisation. They should be combined with effective contraception in women and pregnancy should not be started within 6 months after they are discontinued (risedronate preferred in young women). Teriparatide can be used within the context of its marketing authorisation.

##### 4.4.3.4 Follow-up

- Any use of a specific treatment for osteoporosis beyond 2 years must be reassessed by a specialist.
- Annual monitoring of DXA is recommended for the first 2 years of treatment and when it is discontinued, at a minimum.
- Subclinical fractures should be screened by X-rays of the spine in the case of loss of height of ≥ 2 cm during follow-up or if there is spinal pain.
- The measurement of bone turnover markers is of no interest.
- Discontinuing a specific treatment must be subject to an assessment of the benefit/risk ratio on a case-by-case basis (specialized consultation).

#### 4.4.4 Other measures related to corticosteroid therapy

A corticosteroid therapy increases the risk of infection and can lead to latent infections that should be prevented by vaccination or prophylactic anti-infective treatments.

The vaccines recommended for patients treated with immunosuppressants, biotherapy and/or corticosteroid therapy for a chronic autoimmune or inflammatory disease are the vaccinations of the immunization schedule in effect. In addition, vaccinations against the flu and invasive pneumococcal infections are specifically recommended; vaccination against papillomavirus is suggested for adolescent girls, and even as young as 9 years of age.

It is recommended that vaccinations be updated as early as possible during the autoimmune disease, prior to the start of the immunosuppressant treatment if possible, especially for live attenuated vaccines (yellow fever, varicella zoster, measles, mumps, rubella).

Live attenuated vaccines are theoretically contraindicated in subjects receiving an immunosuppressant therapy, biotherapy and/or a corticosteroid at an immuno-suppressive dose. However, some live attenuated vaccines, particularly against measles (usually measles-mumps-rubella vaccine) or chickenpox, appear to be well tolerated in patients on an immunosuppressant therapy, including biological treatments. In a non-immunized patient, particularly in the context of an epidemic, the benefit/risk balance should be discussed on a case-by-case basis, even when the immunosuppressant treatment cannot be interrupted.

Vaccination against invasive pneumococcal infections should be done with the 13-valent polysaccharide conjugate vaccine in an age-appropriate regimen, followed at least 2 months later by the administration of the 23-valent polysaccharide non-conjugate vaccine (if age > 2 years).

The diphtheria-tetanus-poliomyelitis booster must be given in compliance with the recommendations for the general population. Moreover, vaccination of those in the social circle of these patients, including health care workers, constitutes a major element of protection.

Recent contact with a person with tuberculosis, a history of untreated and spontaneously cured TB, an intradermal reaction to tuberculin of ≥ 10 mm in the absence of a BCG vaccination, or a positive Quantiferon® test should lead to consideration of the treatment of latent tuberculosis in conjunction with the introduction of the corticosteroid therapy. If rifampicin is prescribed, corticosteroid doses should be increased by about 30% to counteract the enzymatic induction effect of rifampicin.

Hyperinfection strongyloidiasis or malignant strongyloidiasis should be prevented by an anti-parasitic eradicator treatment at the time of the introduction of the corticosteroid therapy in any patient who has been in an endemic area (tropical or subtropical regions, southern Europe).

Prevention of the secondary metabolic effects of corticosteroid therapy prescribed for a prolonged time (other than osteoporosis) is another major component for prescribing corticosteroids. Cortico-induced diabetes is common and should be screened for as soon as the corticosteroid therapy is started. The intervention of a dietician should be routinely proposed for implementing a diet adapted to carbohydrate intake, but also to prevent weight gain or sodium and fluid retention through advice on caloric and sodium intake. For the prevention of corticosteroid-induced myopathy, it is recommended that patients perform regular physical activity (fast walking 30–45 min a day), or even to do muscle-strengthening physiotherapy sessions in case of proven amyotrophy.

Resorting to a psychiatrist's advice may be helpful for patients with a psychiatric history in order to assess the risk of psychiatric decompensation when on the corticosteroid therapy.

#### 4.4.5 Therapeutic education

The therapeutic patient education (TPE) is care inseparable from the management of a chronic disease. TPE is a key component of overall patient care. This multidisciplinary approach has been defined by WHO:

"TPE is designed to help patients acquire or maintain the skills they need to best manage their lives with a chronic disease.

It is an integral and permanent part of the patient's care; it includes organized activities, including psychosocial support, designed to make patients aware and informed about their illness, care, hospital organization and procedures, and behaviors related to health and the disease. The purpose of it is to help them (and their families) understand their illness and their treatment, work together and take on their responsibilities for their own care in order to help them maintain and improve their quality of life.

Oral or written information and advice on prevention may be provided by a health professional on a variety of occasions, but they are not the same as therapeutic patient education.”

"The educational approach is participatory and centered on the person and not simply transmitting knowledge or skills."

"This is a partnership relationship between the patient, their social circle and the health care team whose purpose is to help the patient to take care of him/herself."

Thus, TPE gives patients the opportunity to participate in an individualized and controlled health trajectory involving a therapeutic standard proposed by the healthcare team and that of the patient resulting from his/her representations and projects.

There are different ways for health professionals to help their therapeutic education projects. In particular, reference and competence centers, as well as rare disease health networks in particular, are dedicated to providing information. Patient associations and websites can provide useful information.

There is currently no therapeutic patient education program dedicated to Takayasu’s arteritis validated by an ARS [regional health agency]. However, patients with Takayasu’s arteritis may be included in programs for patients with chronic diseases treated with long-term corticosteroid therapy and/or biotherapy.

It appears necessary in this population to suggest an interview with a dietician at the time of a Takayasu’s arteritis diagnosis and the implementation of corticosteroid therapy in order to adapt the diet in a personalized way, if necessary. Caregivers caring for these patients should inform them and their caregivers of the importance of regular corticosteroid therapy, the risk of suddenly discontinuing it and warning signs suggesting a relapse or complications from Takayasu’s arteritis.

#### 4.4.6 Socio-professional, academic and renewable long-term condition aspects

The socio-professional or academic impact of the disease may be significant. Professional reclassification or a disability pension may be necessary. Leave is often essential during the first 6 months of treatment in case of an adjuvant treatment with corticosteroid therapy.

In case of progression towards multiple disabilities, it may be necessary to provide daily life arrangements (home, vehicle, professional reclassification ...) and to pay special attention when filling out the medical portion of the application files with the MDPH.

For school children, it is strongly recommended that an IEP (Individualized Education Program) be established with the school principal.

Due to the duration of the conventional initial treatment (at least 24 months on average) and the prolonged risk of relapse that requires long-term monitoring, the designation of a Long-Term Condition may be assigned for a renewable 5-year period.

#### 4.4.7 Use of patient associations

All health professionals and patients should be informed by their physician of the existence of patient associations, referral and/or competence centers, institutional websites and Orphanet (see *List of useful links for healthcare professionals and patients*).

These associations contribute to better overall management of the disease by promoting cooperation between patients, patient associations, caregivers and medical, social and administrative institutions.

### 4.5 Revascularization treatments

Vascular complications are the main source of morbidity-mortality during Takayasu’s arteritis. Management of these vascular complications is similar to that of common atherosclerosis lesions. The important thing is to avoid intervention on arterial lesions during the inflammatory period, the risk of restenosis being particularly high and potentially multiplied by 7 in this context. This risk of restenosis was 32% in another study, and more common in patients on corticosteroids alone compared to those on an a combined immunosuppressant.

Regular monitoring is necessary to detect the appearance of new arterial lesions, as well as complications such as stenosis, thrombosis or aneurysms. There is no consensus on the procedures and pace of this monitoring, and it must take into account the control of the disease, the existence and location of arterial complications, the age of the patient and the techniques available at each site. Treatment for revascularization of a symptomatic stenosing lesion should be subject to a multidisciplinary discussion by a trained team.

**Box 5.**

**Table:** 

*Situations requiring discussion of a revascularizationor intervention* To the extent that it is possible, when there is no inflammatory phase Stenosis of the renal arteries with drug-resistant renovascular hypertension Symptomatic arterial stenosis with permanent downstream ischemia Aneurysm (depending on its location and size) Indications of revascularization should be routinely discussed with an expert center.	

## 5 Follow-up

Takayasu’s arteritis is a chronic disease that develops through vascular inflammatory flare-ups of which the healing phase most often results in a stenosing lesion of an arterial trunk. Therefore, it requires special follow-up, because nearly 50% of the patients will experience at least one relapse during the course of the disease.

The follow-up involves ensuring the correct therapeutic response to the clinical and biological manifestations of the disease during relapses and cataloging residual lesions (stenosing or aneurysmal) confirmed as established within 3 to 6 months following appropriate therapeutic management.

### 5.1 Objectives

In general, the follow-up will be based on the following 6 points:

- Ensuring proper control of the disease activity and detecting and treating possible relapses of Takayasu’s arteritis
- Ensuring the treatment is reduced in patients whose disease activity is controlled
- Checking tolerance of the treatment
- Detecting early and delayed complications of the disease or its treatments
- Early detection and treatment of the after-effects related to Takayasu’s arteritis or its treatments
- Providing the therapeutic patient education

The main objective is to prevent the occurrence of vascular inflammatory flare-ups of Takayasu’s arteritis, in order to avoid the hemodynamic consequences of a healing process which may be stenosing (claudication, ischemia, necrosis) or more rarely aneurysmal (embolization, thrombosis). The ideal is to prevent the inflammatory flare-up whose first morphological signs may precede the clinical signs.

Pediatric follow-up of Takayasu’s arteritis contains the following features:compliance

- Education workshops devoted to children and parents.
- Adaptation and information regarding the school environment (individualized education plan if necessary).
- Tolerance of treatments with regard to growth.
- Risk related to radiation making an angio-MRI and Doppler ultrasound preferable in the routine follow-up; reserving the arteriography, angio-CT and PET-CT-FDG for situations with therapeutic issues.

**Box 6.**

**Table:** 

*Determining the therapeutic response* No new onset of vascular symptoms No clinical symptoms of inflammation (arthritis, myalgia, scleritis...) or related inflammatory disease No biological inflammatory syndrome Lack of progression on imaging	

### 5.2 Professionals involved (and coordination arrangements)

The follow-up is ideally coordinated by the specialist physician with expertise in Takayasu’s arteritis, usually a vascular physician or an internist or rheumatologist, working together with the attending physician. Other professionals may be involved, such as pediatric doctors (pediatric forms), cardiologists or nephrologists in cases of renovascular hypertension, gastroenterologists in case of associated inflammatory bowel disease; rheumatologists for associated spondyloarthropathy or corticosteroid-induced fractures from osteoporosis, ophthalmologists for specific eye impairment, pulmonologists for PAH, radiologists and vascular surgeons in the event a revascularization procedure is considered. Paramedical disciplines may be called on to intervene: psychologist, child psychologist, dietician, physiotherapist...

### 5.3 Following up on the disease activity

The NIH criteria are usually used to follow up on the activity of Takayasu’s arteritis. They imply that the patient is clinically symptomatic whether in the inflammatory stage of the disease or in the occlusive phase.

The NIH activity criteria are:

Recent onset or worsening of at least 2 of the following criteria:

- Clinical signs of ischemia or vascular inflammation: claudication of a limb, decrease or absence of a pulse, vascular murmur or pain, blood pressure asymmetry.
- Signs of worsening or new vascular lesions on imaging.
- Systemic symptoms: fever, arthromyalgia, episcleritis.
- Biological inflammatory syndrome.

Unfortunately, these criteria do not take into account progress in imaging. It is sometimes possible to intervene therapeutically at a preclinical stage, but these indications must be discussed with an expert center.

### 5.4 Detecting and monitoring complications from the treatment

As with any patient treated with high-dose corticosteroid therapy, this treatment requires monitoring the side effects of corticosteroids, such as diabetes, by regular fasting blood glucose measurements, or high blood pressure, especially by self-measurements of blood pressure or other signs of sodium and water retention and a weight gain. Cramps, hypokalemia, sleep or mood disorders, tremors, psychotic states and aseptic osteonecrosis may also occur. Complications due to infections should be prevented, detected and treated quickly. Any fever should first lead to suspecting an infection. Osteoporosis may require specific monitoring by bone densitometry. Eye complications (cataracts, glaucoma) warrant an ophthalmological follow-up.

During the period of withdrawal from the corticosteroid therapy, there is a risk of corticotropin deficiency that requires a slow and gradual reduction of the drug, starting with the dosage of 7.5 mg/day of prednisone. The practice of routinely replacing prednisone (from 5 mg/day) with hydrocortisone (20–30 mg/day) does not provide a clear advantage over the gradual reduction of prednisone until endogenous cortisone secretion is restored. Some practitioners suggest assessing the response of endogenous cortisone secretion by a corticotropin stimulation test (Synacthen®) at 250 µg. There is no need to maintain corticosteroid replacement therapy (by prednisone or hydrocortisone) in patients with a good response to this test, i.e., about one out of two patients. In all cases, the risk of a corticotropin deficiency in these patients with prolonged corticosteroid therapy should be kept in mind when they find themselves in a stressful situation, such as an intercurrent infection or surgery.

Prescribing methotrexate requires monitoring of the complete blood count, liver function, kidney function and respiratory symptoms that could result in an acute drug-induced lung disease. Patients on methotrexate should be reminded of the risk of fetopathy, and therefore ensure that effective contraception is used. In the case of a pregnancy started while on methotrexate, the drug must be stopped immediately and a specialized consultation in a specialized obstetric setting organized to assess the risk of fetal disease, and the ways to continue or terminate the pregnancy.

Prescribing azathioprine requires monitoring the complete blood count, liver function, kidney function and digestive symptoms that could result in acute drug-induced pancreatitis. The risk of a drug interaction should be checked and prevented, especially with allopurinol, febuxostat and ribavirin. The patient's metabolic status regarding thiopurine methyltransferase should be checked as soon as the treatment begins, or even before. In the case of a slow metabolizing phenotype, the dosage should be reduced so as not to expose the patient to side effects, especially cytopenia. Finally, it is possible to measure the metabolites of azathioprine and 6-thioguanine nucleotides, providing confirmation that there is no underdose in the case of ineffectiveness.

During the follow-up, the use of anti-TNF requires (in addition to the pre-therapeutic assessment) a regular clinical examination looking for signs of heart failure, elements suggesting a malignant or autoimmune pathology, an infectious pathology and signs of allergic-looking skin within hours following the use of anti-TNF.

### 5.4 Detecting vascular complications

An assessment of the inflammatory nature of arterial lesions during follow-up to Takayasu’s arteritis is vital.

All the useful morphological and biological means of examination during diagnosis can be used during the follow-up.

Biological monitoring of the inflammation parameters (inflammatory profile with C-reactive protein, fibrinogen, orosomucoid...) allows early identification of an inflammatory phase, and even a moderate increase in one of these parameters during follow-up justifies looking for a relapse by further examinations.

The Doppler ultrasound test shows regular hypoechoic circumferential thickening of the affected areas and the presence of long, regular stenoses in the acute phase. These signs may improve with treatment but do not always return to normal in the quiescent phase.

In the chronic phase, the CT radiological aspect of Takayasu’s arteritis is less characteristic, and a differential diagnosis with atherosclerosis lesions is difficult. Parietal thickening is often more discrete (2–3 mm) and more irregular than during the acute phase (parietal remodeling), and calcifications may be numerous. There is no significant delayed parietal contrast uptake in principle.

Damage to the pulmonary artery, occurring in 30–50% of the cases, most often occurs during follow-up and may manifest itself by parietal thickening, an aneurysm or stenosis, or even an occlusion viewed on an angio-CT or angio-MRI. Secondary pulmonary perfusion defects can be viewed on an angio-CT or ventilation/perfusion lung scan.

On an angio-MRI examining the aorta and its branches in the quiescent phase of the disease, there are no signs of disease activity using T1 weighted imaging with and without a gadolinium injection and T2 weighted imaging with fat saturation. It is important to note that a parietal edema does not seem to predict the patient's subsequent progress within the limits of the available data.

PET scans are theoretically worthwhile in determining the disease activity and assessing the response to the treatment with greater sensitivity than other imaging methods. However, there is no formal correlation between the avidity for the FDG of the arterial wall and the disease activity. There may be a parietal fixation in the quiescent phases.

Overall, in addition to a multi-year biological assessment, an annual morphological and functional assessment seems necessary for the progressive follow-up of Takayasu’s arteritis: the combination of at least a Doppler ultrasound and an angio-CT/MRI seems pertinent to us. The routine use of the PET scan is debatable, since this test seems to have greater sensitivity than other tests for identifying activity in the vascular wall, but low specificity.

Given the consequences of the onset of a new vascular lesion, the presence of signs of progression of the disease on a test must be confirmed by another imaging type, and therapeutic intensification discussed.

**Box 7.**

**Table:** 

*Suggested sequence for monitoring the imaging* Focus on non-radiation imaging methods if there are no new symptoms. After starting an initial treatment: between 3 and 6 months In the inactive phase: between 6 and 12 months	

### 5.5 Screening for cardiovascular complications

Myocardial impairment is rare, often subclinical. If an MRI is used as a vascular exploration test, supplementing it with a reference myocardial exploration at the same time may be considered. However, a routine cardiac MRI in the absence of clinical, electrical or biological signs of myocardial impairment appears to be more debatable.

Looking for valve impairment by echocardiography, in particular aortic insufficiency, should be routine, because it occurs frequently.

In the case of progressive thoracic aortitis, coronary ostial disease is possible and requires investigation by questioning, clinical examination, an ECG and possibly a coronary CT angiography.

The search for high blood pressure is essential because it occurs often. Its various etiologies determine the tests to be performed: search for stenosis of the renal arteries, descending aortic stenosis resulting in aortic pseudo-coarctation. In order not to underestimate high blood pressure, the search for a subclavian or axillary arterial lesion is necessary. Checking blood pressure at the ankles may be an alternative in the absence of a lesion on the aorta or its branches supplying the lower limbs.

## 6 Takayasu’s arteritis and pregnancy

There is little data during the course of a pregnancy, although this pathology mainly affects young women of childbearing age. Pregnancy, however, appears to be associated with significant maternal and fetal morbidity-mortality during Takayasu’s arteritis. Possible complications include hypertensive flare-ups, ischemic events (stroke), fetal (fetal loss, growth retardation in utero, prematurity) and maternal (pre-eclampsia) obstetric complications.

The activity of Takayasu’s arteritis increases this obstetric risk.

Management of these pregnancies is optimized by planning for them, ideally during a pre-conception consultation and by multidisciplinary management by trained teams, with at least monthly consultations within the context of "at risk pregnancies".

### 6.1 Impact of Takayasu’s arteritis on pregnancy

The overall rate of spontaneous miscarriages (spontaneous abortion before 22 weeks of amenorrhea) of Takayasu’s arteritis patients is about 10% of pregnancies and does not appear to be different from that of the general population.

The rate of prematurity (birth before 37 weeks of amenorrhea) in patients with Takayasu’s arteritis is 8% compared to 7% in the general population.

The proportion of children with intrauterine growth retardation (IUGR), weight < 10th percentile for the pregnancy term, is about 5% compared to 1–2% in the general population. Regular biometric ultrasound monitoring during pregnancy is therefore recommended.

There does not appear to be any difference in peri-natal morbidity-mortality in women with Takayasu’s arteritis.

The rate of pre-eclampsia/eclampsia in patients with Takayasu’s arteritis is about 20% of pregnancies

Takayasu’s arteritis increases the risk of obstetric complications by more than 10. pBlood pressure should be very diligently monitored during any pregnancy in a patient with Takayasu’s arteritis.

### 6.2 Impact of pregnancy on Takayasu’s arteritis

Pregnancy appears to increase the risk of developing or worsening high blood pressure in patients with Takayasu’s arteritis. During pregnancy, 25% of women with Takayasu’s disease have either de novo hypertension or a worsening of their high blood pressure.

### 6.3 Management during pregnancy

#### 6.3.1 Pre-conception consultation

Planning for a pregnancy in a patient with Takayasu’s arteritis during a pre-conception consultation should be considered. The primary purpose of this consultation is to determine the contraindications to pregnancy (heart failure, severe or uncontrolled high blood pressure, progressive vascular lesion). It makes it possible to adapt the primary treatment (stopping a converting enzyme inhibitor (CEI), replacing a teratogenic immunosuppressant with azathioprine, which is authorized during pregnancy). CEIs and angiotensin II receptor antagonists (ARA2) are contraindicated in the second and third trimesters of pregnancy and are not recommended in the first. During the pre-conception consultation, it is therefore advisable to change the treatment and consider a therapeutic alternative. The use of calcium inhibitors for high blood pressure and vascular disease is allowed (see the website of the Teratogenic Agent Reference Center—www.lecrat.fr). It is important to make sure that the patient is immunized against rubella, vaccinate her if necessary (if there are no contraindications), update the other vaccinations and start folic acid supplements. Low-dose aspirin is allowed, but is not routine and depends on the vascular impairment. The use of oral corticosteroids (prednisone) should not exceed 10–15 mg/d. A complete clinical and paraclinical assessment before the start of pregnancy is advisable: cardiovascular assessment, reference vascular assessment (features and severity of the vascular lesions), complete biological assessment (CRP, thyroid function, vitamin deficiencies).

#### 6.3.2 During pregnancy

Takayasu’s arteritis should be as quiescent as possible. Signs of Takayasu’s arteritis activity is related to a poor obstetric and maternal prognosis primarily associated with high blood pressure.

In addition to monthly gynecological monitoring (fetal biometrics, uterine and umbilical Dopplers), signs of disease activity must be looked for. It therefore includes, on a monthly basis:

- At the clinical level:
  - Blood pressure and urinary dipstick, essential for pre-eclampsia or eclampsia.
  - Look for signs of a progression of Takayasu’s arteritis: carotidodynia, claudication of a limb, signs of heart failure, neurological manifestation...
  - Look for signs of obstetric complications: abdominal pain, especially band-like epigastric pain, swelling of the lower limbs...

- At the biological level:
  - Complete blood count;
  - CRP;
  - Serum creatinine level, proteinuria;
  - Transaminases, gamma GT;
  - Glycemia, toxoplasmosis and rubella serologies, if negative.

- At the ultrasound level:

In addition to the 3 mandatory fetal ultrasounds (12 weeks of amenorrhea, 22 weeks of amenorrhea, 32 weeks of amenorrhea), it will be suggested that this test be repeated more often, especially in patients with high blood pressure, or if there is an obstetric history. These ultrasounds will be supplemented by uterine and umbilical Dopplers from 22 weeks of amenorrhea.

Treatment during pregnancy should help control Takayasu's arteritis while being compatible with a good progression of the pregnancy. If an immunosuppressant treatment is required to control Takayasu’s arteritis during pregnancy, azathioprine is allowed (CRAT [Teratogenic Agent Reference Center] website: www.lecrat.fr). Azathioprine at a dose of 2 mg/kg can be used in the case of pregnancy to replace other immunosuppressants that are teratogenic. The use (or continuation) of infliximab will only be considered after other possible treatment options (corticosteroids, azathioprine) have been ruled out. If the use of infliximab is indispensable, to the extent it is possible, schedule a final administration at the beginning of the third trimester because of its long elimination half-life. Children whose mothers have been treated with infliximab during pregnancy are considered immunosuppressed for six months after the last maternal injection (including fetal life).

#### 6.3.3 Childbirth and postpartum

Aspirin is not considered to be contraindicated for epidural analgesia, but it may be discontinued at 35 weeks of amenorrhea.

#### 6.3.4 Breastfeeding

Breastfeeding is most often possible, provided that the treatments are compatible (CRAT website: www.lecrat.fr). Aspirin used in a platelet antiaggregant dose is compatible with breastfeeding. Prednisone is also compatible with breastfeeding. Breastfeeding is possible during a treatment with azathioprine or infliximab. As for methotrexate, it is best to wait 24 hours after taking it to breastfeed a child. Significant concentrations of cyclophosphamide are found in breast milk, and breastfeeding is contraindicated when taking this treatment.

*List of useful links for health professionals and patients*

Information for health professionals:

- FAI^2^R—Health network of rare autoimmune and auto-inflammatory diseases, www.fai2r.org.
- FAVA-Multi—Network of rare vascular diseases with systemic impairment, www.favamulti.fr.
- Orphanet—www.orpha.net.

Information for patients:

- Rare diseases alliance—www.alliance-maladies-rares.org.
- EURORDIS—Federation of associations of patients and individuals active in the field of rare diseases, www.eurordis.org.
- Association Takayasu France.
- France Vasculitis Association—www.association-vascularites.org.
- FAI^2^R—Health network of rare autoimmune and auto-inflammatory diseases, www.fai2r.org.
- FAVA-Multi—Health network of rare vascular diseases with systemic impairment, www.favamulti.fr.
- Rare Diseases Info Services—www.maladiesraresinfo.org.

## Data Availability

Not applicable.

## Bibliography

- Aeschlimann FA, Eng SWM, Sheikh S, Laxer RM, Hebert D, Noone D, et al. Childhood Takayasu arteritis: disease course and response to therapy. Arthritis Res Ther [Internet]. 2017;19(1).
- Brunner J, Feldman BM, Tyrrell PN, Kuemmerle-Deschner JB, Zimmerhackl LB, Gassner I, et al. Takayasu arteritis in children and adolescents. Rheumatology. 2010;49(10):1806–14.
- Arend WP, Michel BA, Bloch DA, Hunder GG, Calabrese LH, Edworthy SM, et al. The American College of Rheumatology 1990 criteria for the classification of takayasu arteritis. Arthritis Rheum. 2010;33(8):1129–34.
- Arnaud L, Haroche J, Malek Z, Archambaud F, Gambotti L, Grimon G, et al. Is 18 F-fluorodeoxyglucose positron emission tomography scanning a reliable way to assess disease activity in takayasu arteritis? Arthritis Rheum. 2009;60(4):1193–200.
- Arnaud L, Haroche J, Limal N, Toledano D, Gambotti L, Chalumeau NC, et al. Takayasu Arteritis in France: A Single-Center Retrospective Study of 82 Cases Comparing White, North African, and Black Patients. Medicine (Baltimore). 2010;89(1):1–17.
- Aydin SZ, Yilmaz N, Akar S, Aksu K, Kamali S, Yucel E, et al. Assessment of disease activity and progression in Takayasu’s arteritis with Disease Extent Index-Takayasu. Rheumatology. 2010;49(10):1889–93.
- Batu ED, Sönmez HE, Hazırolan T, Özaltın F, Bilginer Y, Özen S. Tocilizumab treatment in childhood Takayasu arteritis: Case series of four patients and systematic review of the literature. Semin Arthritis Rheum. 2017;46(4):529–35.
- Brunner J, Feldman BM, Tyrrell PN, Kuemmerle-Deschner JB, Zimmerhackl LB, Gassner I, et al. Takayasu arteritis in children and adolescents. Rheumatology. 2010;49(10):1806–14.
- Choe YH, Han B-K, Koh E-M, Kim D-K, Do YS, Lee WR. Takayasu’s Arteritis: Assessment of Disease Activity with Contrast-Enhanced MR Imaging. Am J Roentgenol. 2000;175(2):505–11.
- Comarmond C, Cluzel P, Toledano D, Costedoat-Chalumeau N, Isnard R, Gaudric J, et al. Findings of Cardiac Magnetic Resonance Imaging in Asymptomatic Myocardial Ischemic Disease in Takayasu Arteritis. Am J Cardiol. 2014;113(5):881–7.
- Comarmond C, Dessault O, Devaux J-Y, Costedoat-Chalumeau N, Resche-Rigon M, Isnard R, et al. Myocardial Perfusion Imaging in Takayasu Arteritis. J Rheumatol. 2013;40(12):2052–60.
- Comarmond C, Mirault T, Biard L, Nizard J, Lambert M, Wechsler B, et al. Takayasu Arteritis and Pregnancy. Arthritis Rheumatol. 2015;67(12):3262–9.
- Comarmond C, Biard L, Lambert M, Mekinian A, Ferfar Y, Kahn J-E, et al. Long-Term Outcomes and Prognostic Factors of Complications in Takayasu Arteritis: A Multicenter Study of 318 Patients. Circulation. 2017;136(12):1114–22.
- Dagna L, Salvo F, Tiraboschi M, Bozzolo EP, Franchini S, Doglioni C, et al. Pentraxin-3 as a Marker of Disease Activity in Takayasu Arteritis. Ann Intern Med. 2011;155(7):425.
- Danda D, Goel R, Joseph G, Kumar ST, Nair A, Ravindran R, et al. Clinical course of 602 patients with Takayasu's arteritis: comparison between Childhood-onset versus adult onset disease. Rheumatology (Oxford). 2020 Nov 12:keaa569. https://doi.org/10.1093/rheumatology/keaa569.
- Dejaco C, Ramiro S, Duftner C, Besson FL, Bley TA, Blockmans D, et al. EULAR recommendations for the use of imaging in large vessel vasculitis in clinical practice. Ann Rheum Dis. mai 2018;77(5):636–43.
- Fiessinger JN, Tawfik-Taher S, Capron L, Laurian C, Cormier JM, Camilleri JP, et al. [Takayasu’s disease. Diagnostic criteria (author’s transl)]. Nouv Presse Med. 1982;11(8):583–6.
- Gribbons KB, Ponte C, Carette S, Craven A, Cuthbertson D, Hoffman GS, et al. Patterns of Arterial Disease in Takayasu Arteritis and Giant Cell Arteritis. Arthritis Care Res (Hoboken). 2020 Nov;72(11):1615-1624.
- Hellmich B, Agueda A, Monti S, Buttgereit F, de Boysson H, Brouwer E, et al. 2018 Update of the EULAR recommendations for the management of large vessel vasculitis. Ann Rheum Dis. 2019;annrheumdis-2019-215672.
- Ishikawa K. Diagnostic approach and proposed criteria for the clinical diagnosis of Takayasu’s arteriopathy. J Am Coll Cardiol. 1988;12(4):964–72.
- Kato Y, Terashima M, Ohigashi H, Tezuka D, Ashikaga T, Hirao K, et al. Vessel Wall Inflammation of Takayasu Arteritis Detected by Contrast-Enhanced Magnetic Resonance Imaging: Association with Disease Distribution and Activity. Hosoda T, éditeur. PLOS ONE. 2015;10(12):e0145855.
- Kerr GS. Takayasu Arteritis. Ann Intern Med. 1994;120(11):919.
- Li J, Wang Y, Wang Y, Wang Y, Yang Y, Zhao J, et al. Association between acute phase reactants, interleukin-6, tumor necrosis factor-α, and disease activity in Takayasu's arteritis patients. Arthritis Res Ther. 2020 Dec 10;22(1):285.
- Mach F, Baigent C, Catapano AL, Koskinas KC, Casula M, Badimon L, et al. 2019 ESC/EAS Guidelines for the management of dyslipidaemias: lipid modification to reduce cardiovascular risk: The Task Force for the management of dyslipidaemias of the European Society of Cardiology (ESC) and European Atherosclerosis Society (EAS). Eur Heart J. 2019. https://doi.org/10.1093/eurheartj/ehz455.
- Mekinian A, Lambert M, Huglo D, Devos P, Mirault T, Steinling M, et al. Pulmonary perfusion scintigraphy: A tool to detect the presence of pulmonary artery involvement in Takayasu’s arteritis. Presse Médicale. 2012;41(2):e37–42.
- Mekinian A, Comarmond C, Resche-Rigon M, Mirault T, Kahn JE, Lambert M, et al. Efficacy of Biological-Targeted Treatments in Takayasu Arteritis: Multicenter, Retrospective Study of 49 Patients. Circulation. 2015;132(18):1693–700.
- Moriwaki R, Noda M, Yajima M, Sharma BK, Numano F. Clinical Manifestations of Takayasu Arteritis in India and Japan—New Classification of Angiographic Findings. Angiology. 1997;48(5):369–79.
- Nakaoka Y, Isobe M, Takei S, Tanaka Y, Ishii T, Yokota S, et al. Efficacy and safety of tocilizumab in patients with refractory Takayasu arteritis: results from a randomised, double-blind, placebo-controlled, phase 3 trial in Japan (the TAKT study). Ann Rheum Dis. 2018;77(3):348–54.
- Numano F. The story of Takayasu arteritis. Rheumatology. janv 2002;41(1):103–6.
- Piepoli MF, Hoes AW, Agewall S, Albus C, Brotons C, Catapano AL, et al. 2016 European Guidelines on cardiovascular disease prevention in clinical practice: The Sixth Joint Task Force of the European Society of Cardiology and Other Societies on Cardiovascular Disease Prevention in Clinical Practice (constituted by representatives of 10 societies and by invited experts) Developed with the special contribution of the European Association for Cardiovascular Prevention & Rehabilitation (EACPR). Eur Heart J. 2016;37(29):2315–81.
- Ruperto N, Ozen S, Pistorio A, Dolezalova P, Brogan P, Cabral DA, et al. EULAR/PRINTO/PRES criteria for Henoch-Schonlein purpura, childhood polyarteritis nodosa, childhood Wegener granulomatosis and childhood Takayasu arteritis: Ankara 2008. Part I: Overall methodology and clinical characterisation. Ann Rheum Dis. 2010;69(5):790–7.
- Saadoun D, Lambert M, Mirault T, Resche-Rigon M, Koskas F, Cluzel P, et al. Retrospective Analysis of Surgery Versus Endovascular Intervention in Takayasu Arteritis: A Multicenter Experience. Circulation. 2012;125(6):813–9.
- Schmidt J, Kermani TA, Kirstin Bacani A, Crowson CS, Matteson EL, Warrington KJ. Tumor necrosis factor inhibitors in patients with Takayasu arteritis: Experience from a referral center with long-term follow-up. Arthritis Care Res. 2012.
- Soto ME, Espinola N, Flores-Suarez LF, Reyes PA. Takayasu arteritis: clinical features in 110 Mexican Mestizo patients and cardiovascular impact on survival and prognosis. Clin Exp Rheumatol. 2008;26(3 Suppl 49):S9-15.
- Soussan M, Nicolas P, Schramm C, Katsahian S, Pop G, Fain O, et al. Management of Large-Vessel Vasculitis With FDG-PET: A Systematic Literature Review and Meta-Analysis. Medicine (Baltimore). 2015;94(14):e622.
- Williams B, Mancia G, Spiering W, Agabiti Rosei E, Azizi M, Burnier M, et al. 2018 ESC/ESH Guidelines for the management of arterial hypertension. Eur Heart J. 2018;39:3021–104.

## Abbreviations

ACR: American College of Rheumatology
LTC: Long-term condition
AM: Authorization to market
RHA: Regional health agency
CVA: Stroke, cerebrovascular accident
BAFF: B-cell activating factor
BCG: Bacillus Calmette–Guerin
CRP: C-reactive protein
DXA: Bone densitometry
ECG: Electrocardiogram
FDG: Fluorodeoxyglucose
HBP: High blood pressure
ACE: Angiotensin-converting enzyme inhibitors
IL-6: Interleukin 6
MRI: Magnetic resonance imaging
ABPM: Ambulatory blood pressure measurement
MDPH: Departmental home for persons with disabilities
NIH: National Institute of Health
IRP: Individualized reception project
PNDS: National diagnostic and care protocol
IUGR: Intrauterine growth retardation
WA: Weeks of amenorrhea
SMR: Standardized mortality rate
CT: Computerized tomography scan
TNFα: Tumor necrosis factor alpha
ESR: Erythrocyte sedimentation rate

